# Cardiac Index by Transthoracic Echocardiography (CITE) study

**DOI:** 10.1371/journal.pone.0207269

**Published:** 2018-12-12

**Authors:** Barna Szabó, Eszter Krisztina Marosi, Katarina Vargová, Noémi Nyolczas

**Affiliations:** 1 Heart-Lung-Physiology Clinic, Örebro University Hospital, Örebro, Sweden; 2 Cardiology, Military Hospital State Health Centre, Budapest, Hungary; The University of Hong Kong, HONG KONG

## Abstract

**Aims:**

Left ventricular ejection fraction (LVEF) is the most frequently used parameter in the assessment of heart failure (HF). Cardiac index (CI) is considered a potential alternative to LVEF despite limited evidence. We aimed to assess and compare the predictive accuracy of LVEF and echocardiographically-assessed CI in HF patients.

**Methods and results:**

A single-centre, retrospective cohort study was conducted in patients hospitalized for acute HF from 2010–2016. Cox proportional hazard models including either LVEF or CI were created to predict all cause death, cardiovascular (CV) death, or first HF-readmission. Of 334 patients included in the analysis, 58.7% exhibited HF with reduced LVEF (HFrEF). Left ventricular ejection fraction did not show correlation with any endpoint, while CI was predictive of HF-readmission in the entire cohort. Both the LVEF-based and CI-based models demonstrated moderate discriminative accuracy when predicting all-cause death, CV death, or HF-readmission. Left ventricular ejection fraction proved to be an independent predictor of CV mortality in HFrEF-patients, while CI was predictive of HF-readmission in the non-HFrEF group.

**Conclusions:**

Left ventricular ejection fraction seemed to be associated more closely with disease severity in HFrEF, and CI in the non-HFrEF group, in this real-life cohort of elderly HF patients. The LVEF-based and CI-based predictive models have clinically similar predictive accuracy for mortality and HF-readmission, thus CI may be a potential alternative to LVEF in the assessment of left ventricular function. Cardiac index may be an important new tool in the assessment of HF patients with midrange or preserved LVEF.

## Introduction

Heart failure (HF) affects a large segment of the population worldwide, and has a mortality risk comparable to malignancies.[1, 2] The disease confers a high economic burden on healthcare systems. Efforts have been made to develop prognostic tools and find appropriate measures to select patients who will respond to specific HF treatments. Predictive tools are necessary to accurately identify patients who will benefit from costly advanced treatments of limited availability such as heart transplantation and ventricular assist devices,[3, 4] as well as patients who should receive end of life care.

Early studies identified left ventricular ejection fraction (LVEF) as a useful, easily obtainable, prognostic echocardiographic parameter. Low LVEF is correlated to reduced cardiac output (CO), cardiac index (CI), and poor tissue perfusion and has been shown correlated with increased risk of mortality and morbidity.[5, 6] These correlations have led to widespread use of LVEF as a prognostic tool and as an inclusion criterion in the design of nearly all large-scale HF trials. Although it is a rapid and simple measurement, the accurate assessment of LVEF is challenging, with high intra- and inter-observer variability.[7] Assessment of LVEF is frequently limited by suboptimal image quality in some left ventricular wall segments, variation in left ventricular filling during atrial fibrillation, or by dyssynchronous left ventricular contraction.

Cardiac output and cardiac index have also been shown to correlate with mortality,[8] but their calculation required complex, invasive hemodynamic measurements and in recent decades was largely replaced by LVEF. Currently, CO and CI can be measured noninvasively by transthoracic echocardiography, which has comparable accuracy to the invasive method.[9] However, inaccuracy in the assessment of the size of the left ventricular outflow tract (LVOT) or angle deviation in the measurement of LVOT flow velocities may lead to measurement error. Although we have no available data comparing prognostic value of echocardiographically-assessed CO or CI to that of LVEF, they are considered alternatives to LVEF measurement in the assessment of the severity of HF.[10]

The current research aims to compare the predictive value of LVEF to that of CI-based models for mortality and re-hospitalization in an unselected HF population in a retrospective study.

## Methods

We conducted a retrospective cohort study comprising patients hospitalized for HF from 2010–2016 at the Heart-Lung Clinic of Örebro University Hospital, Sweden. Patients were eligible for inclusion if the primary reason for hospital admission was acute HF, if they had an echocardiography after admission with stored images available for post-hoc measurement of CI, and were at least 18 years old at the time of echocardiography. If a patient had more than one admission matching the inclusion criteria during the study period, only the first such event was taken into account. Patients were excluded if they presented any cardiac condition that would affect the measurement of CI, for example moderate or severe aortic valve disease, hypertrophic obstructive cardiomyopathy or congenital heart disease, or if they did not fulfil the diagnostic criteria of HF according to the 2016 guidelines of the European Society of Cardiology (ESC).[11] No criteria were set for echocardiographic image quality.

All echocardiographic examinations were performed and analysed by experienced echocardiographers. Left ventricular ejection fraction was assessed during initial examination, and a different echocardiographer assessed CI values retrospectively, blinded to outcomes. Cardiac index was calculated as *CI* = *LVOT* − *VTI* * *HR* * *LVOTd*^2^ * 0.7854/*BSA*. Left ventricular outflow tract diameter (LVOTd) was measured from parasternal long axis images, as close to the aortic valve as possible. Left ventricular outflow tract velocity-time integral (LVOT-VTI) was measured on pulsatile doppler LVOT flow pattern images obtained from an apical 5-chamber view. LVOT-VTI was assessed on as many cardiac cycles as possible (maximum of 5) and the average of these measurements was used in the calculation of CI, in order to reduce possible inaccuracies caused by arrhythmias. Measurement bias of LVOTd and LVOT-VTI due to potentially suboptimal imaging plane and angle deviation could however not be eliminated due to the retrospective setting of the study. Mean cardiac cycle length (CCL) was assessed by measuring and averaging the duration of the cardiac cycle of LVOT-VTI measurements from the end of the ejection period of the previous systole to the end of the index ejection period. Heart rate (HR) was then calculated as *HR* = 1/*CCL*. Body surface area (BSA) was calculated using the DuBois-formula.

Endpoints were defined as all-cause death, cardiovascular (CV) death, or first HF-related hospital readmission. Information on HF-related readmission was obtained from electronic patient journals. Mortality and date of death were obtained from the Swedish Death Registry. Cause of death was assessed by two independent investigators based on available patient journals and, in cases of discrepancy, adjudicated by a third.

Prognostic models were created using the Cox proportional hazard method including either LVEF or CI together with available traditional risk factors for HF: age, sex, diabetes, ischemic aetiology, chronic obstructive pulmonary disease (COPD), glomerular filtration rate (GFR), body mass index (BMI), atrial fibrillation, and heart rate. Data of traditional risk factors for morbidity and mortality with HF were collected by manual search in patient electronic journals. The diagnosis of heart failure was confirmed using the diagnostic criteria for heart failure with reduced ejection fraction (HFrEF, LVEF<40%), mid-range ejection fraction (HFmrEF, LVEF between 40–49%) and preserved ejection fraction (HFpEF, LVEF≥50%) as described in the 2016 guidelines of the ESC.[11] Clinical symptoms together with an LVEF below 40% confirmed the diagnosis of HFrEF. In case of clinical symptoms consistent with heart failure but an LVEF of 40% or higher, additional echocardiographic abnormalities (left atrial enlargement and/or left ventricular hypertrophy and/or left ventricular diastolic dysfunction defined as E/Ea≥13) as well as elevated natriuretic peptide levels (BNP above 100pg/ml or NTproBNP above 300pg/ml according to the cut-off limit for acute heart failure) was required to confirm diagnosis.

Categorical variables were compared using chi-square test and unpaired two-tailed t-test was used for the comparison of continuous variables. Discriminative accuracy of the prediction models was assessed by Harrell’s c-index, with standard deviation obtained by the jack-knife method and compared by unpaired t-test. The P-value of 0.05 was considered significant.

The investigation conforms to the principles outlined in the Declaration of Helsinki and was approved by the Regional Ethical Review Board in Uppsala, Sweden. Data on patient demographics and outcomes were collected from the patients’ electronic medical records and the Swedish Death Registry, and linked to echocardiographic data by personal identification numbers. All data was anonymized before further processing. The Regional Ethical Review Board did not waive any requirement for informed consent.

## Results

We found 1823 acute HF-related hospital admissions of 1274 individuals during the study period. Lack of echocardiography after hospital admission excluded 456 patients from the study, and 139 were excluded due to the presence of moderate to severe aortic valve disease or hypertrophic obstructive cardiomyopathy. Of the remaining 679 patients, echocardiography images necessary for CO measurement and documented height and weight needed for BSA calculation at the time of echocardiography were available for 339. Five patients exhibited LVEF≥40% and elevated natriuretic peptides but presented no echocardiographic evidence of diastolic dysfunction or structural heart disease, so did not fulfil the diagnostic criteria of HF. The final subject group of 334 patients was a heterogeneous population comprising patients with reduced ejection fraction (HFrEF) (n = 196), mid-range ejection faction (HFmrEF) (n = 65), or preserved ejection fraction (HFpEF) (n = 73) and included those with newly diagnosed HF before treatment optimization as well as patients with a long history of HF, already on optimal medical treatment at the time of admission. Patient baseline characteristics are shown in Table 1. The mean LVEF was 35.7% and mean CI 2.17 L/min/m^2^. The majority of subjects were >70 years old and had multiple comorbidities. All patients received guideline directed medical treatment. Medications and devices applied after the index hospital admission and after treatment optimization are shown in Table 2. There was no significant difference in any available demographic, clinical parameters or drug treatment between the 334 patients included in the analysis and the 340 who were excluded due to missing echocardiographic images, height or weight data.

**Table 1:** Subject demographics and clinical characteristics.

	Overall cohort	HFrEF	HFmrEF and HFpEF
N	334	196	138
Age (years)	75.8±11.0	73.8±11.4	78.5±9.9
Male	218 (65.3%)	140 (71.4%)	78 (56.5%)
Ischemic aetiology	165 (49.4%)	103 (52.6%)	62 (44.9%)
Diabetes	106 (31.7%)	65 (33.2%)	41 (29.7%)
COPD	47 (14.1%)	23 (11.7%)	24 (17.4%)
GFR-EPI (mL/min/1.73 m^2^)	55.8±25.2	57.2±25.7	53.9±24.4
Atrial fibrillation/flutter	143 (42.8%)	79 (40.3%)	64 (46.4%)
LVEF (%)	35.7±14.7	25.3±7.8	50.4±8.3
LVEF ≥50%	73 (21.9%)	-	73 (52.9%)
CI (L/min/m^2^)	2.17±0.73	2.09±0.72	2.78±0.72
Heart rate (min^-1^)	78.0±17.8	81.5±17.9	73.1±16.5
BMI (kg/m^2^)	27.9±5.6	28.0±5.5	27.8±5.8
BSA (m^2^)	1.94±0.24	1.96±0.23	1.91±0.26

COPD: chronic obstructive pulmonary disease; GFR-EPI: glomerular filtration rate estimated by the EPI-method; LVEF: left ventricular ejection fraction; CI: cardiac index; BMI: body mass index; BSA: body surface area; HFrEF: heart failure with reduced ejection fraction; HFmrEF: heart failure with mid-range ejection fraction; HFpEF: heart failure with preserved ejection fraction

**Table 2:** Medications and devices applied during follow-up, after treatment optimization.

	Overall cohort, n (%)	HFrEF, n (%)	HFmrEF and HFpEF, n (%)
ACEi/ARB	289 (86.5)	182 (92.9)	107 (77.5)
Beta receptor blocker	290 (86.8)	181 (92.3)	109 (79.0)
MRA	153 (45.8)	100 (51.0)	53 (38.4)
Diuretics	283 (84.7)	170 (86.7)	113 (81.9)
CRT	34 (10.2)	28 (14.3)	6 (4.3)^†^
ICD	33 (9.9)	30 (15.3)	3 (2.2)^‡^

ACEi: angiotensin converting enzyme inhibitor; ARB: angiotensin receptor blocker; MRA: mineralocorticoid receptor antagonist; CRT: cardiac resynchronization therapy; ICD: implantable cardioverter defibrillator; HFrEF: heart failure with reduced ejection fraction; HFmrEF: heart failure with mid-range ejection fraction; HFpEF: heart failure with preserved ejection fraction

^†^ All patients had echocardiography showing LVEF ≤35% during follow-up, before CRT-implantation and after the index echocardiography.

^‡^ One patient had an echocardiography with LVEF ≤35% during follow-up, before ICD-implantation and after the index echocardiography. Two patients received secondary prevention ICD.

A total of 1133 cardiac cycles were analysed. Patients with atrial fibrillation had more cardiac cycles available than those with sinus rhythm (3.55±1.21 vs. 3.28±1.19 per patient, p = 0.044). They had significantly lower stroke volume index (SVI = 26.6±8.9 ml/m^2^ vs. 30.5±11.3 ml/ m^2^, p = 0.0005) and higher heart rate (82.0±19.5 vs. 75.0±15.8 beat/min, p = 0.0006), resulting in lower CI (2.10±0.74 vs. 2.23±0.79 l/min/m^2^, p = 0.116); however this difference did not reach statistical significance. As irregular heart rhythm leads to beat-to-beat changes in SVI and CI, intra-observer variability assessment was limited to the subgroup of 179 patients with sinus rhythm and at least 2 cardiac cycles. The absolute intra-observer variability for SVI was 0.2±3.9ml/m^2^ (relative variability 0.5±13.0%). For single beat estimates of CI, intra-observer variability was 0.01±0.24 l/min/m^2^ (relative variability 0.1±10.7%).

One-hundred-seventy-three patients died (92 CV death, 81 non-CV death), and 133 underwent HF-related hospital readmission during 2.3±1.9 years of follow-up. On univariate Cox proportional hazards analysis, LVEF was not associated with all-cause death, CV death, or HF-readmission (*P* = 0.175, *P* = 0.422, and *P* = 0.175 respectively). Cardiac index was not associated with all-cause death (*P* = 0.271) or CV death (*P* = 0.209) but showed significant association with HF-readmission (*P* = 0.020).

When adjusted for traditional risk factors, LVEF showed no significant correlation with the endpoints (*P* = 0.744 for all-cause death, *P* = 0.116 for CV death, *P* = 0.495 for HF-readmission). Cardiac index showed no correlation with all-cause or CV death but remained an independent predictor of HF-readmission (*P* = 0.350, *P* = 0.260, and *P* = 0.016, respectively). The risk ratios of the final prediction models in the overall cohort are presented in Table 3.

**Table 3:** Results of the multivariate Cox analyses in the entire cohort.

	Endpoint		
Risk factor	All-cause death	CV death	HF-readmission
Age (year)	1.060 (1.039–1.081)^†^	1.073 (1.043–1.103)^†^	1.021 (1.000–1.042)
Ischemic aetiology	1.409 (1.027–1.931)^‡^	1.808 (1.158–2.823)^†^	1.537 (1.067–2.215)^‡^
Male	1.435 (1.004–2.051)^‡^	1.553 (0.945–2.552)	1.133 (0.766–1.677)
COPD	1.438 (0.943–2.194)	1.277 (0.702–2.323)	1.824 (1.170–2.844)^†^
Diabetes	1.109 (0.797–1.544)	0.742 (0.460–1.196)	1.015 (0.692–1.489)
Atrial fibrillation	1.130 (0.820–1.558)	0.974 (0.626–1.515)	1.104 (0.765–1.591)
GFR-EPI (mL/min/1.73 m^2^)	0.987 (0.980–0.994)^†^	0.991 (0.981–1.002)	0.985 (0.977–0.993)^†^
Heart rate (min^-1^)	1.004 (0.993–1.014)	1.007 (0.993–1.021)	1.006 (0.995–1.017)
BMI	0.990 (0.957–1.024)	1.021 (0.976–1.067)	1.002 (0.969–1.036)
LVEF (%)	0.999 (0.987–1.012)	0.989 (0.972–1.005)	1.012 (0.998–1.026)
CI (L/min/m^2^)	0.907 (0.731–1.126)	0.882 (0.644–1.207)	0.691 (0.532–0.898)^†^

COPD: chronic obstructive pulmonary disease; GFR-EPI: glomerular filtration rate estimated by the EPI-method; BMI: body mass index; LVEF: left ventricular ejection fraction; CI: cardiac index; CV: cardiovascular; HF: heart failure

^†^ *P* < 0.01

^‡^ *P* < 0.05

Results are presented as risk ratio (95% confidence interval).

Both the LVEF-based and CI-based models had moderate discriminative accuracy when predicting all-cause death (c-index 0.6974 vs. 0.6983, *P* < 0.001), CV death (c-index 0.7270 vs. 0.7255, *P* < 0.001), and HF-readmission (c-index 0.6751 vs. 0.6902, *P* < 0.001), with slight but significant difference in all cases.

### Analysis of subgroups with respect to LVEF

No correlation was found between LVEF and any endpoint with unadjusted analysis in patients with HFrEF (n = 196). Cardiac index showed a strong association with all-cause death but did not demonstrate correlation with CV death or HF-readmission. After adjustment for other risk factors, neither LVEF nor CI showed correlation with all-cause death or HF-readmission, but LVEF became an independent predictor of CV death (Table 4).

**Table 4:** Results of the adjusted and unadjusted multivariate Cox analyses in patients with HFrEF.

	Unadjusted		Adjusted	
	Risk ratio^†^	*P*-value	Risk ratio^†^	*P*-value
All-cause death				
LVEF	1.001 (0.975–1.028)	0.935	0.985 (0.958–1.013)	0.290
CI	0.727 (0.541–0.978)	0.035	0.736 (0.534–1.014)	0.060
CV death				
LVEF	0.985 (0.949–1.022)	0.407	0.959 (0.924–0.995)	0.026
CI	0.817 (0.547–1.221)	0.324	0.726 (0.470–1.121)	0.149
HF-readmission				
LVEF	0.998 (0.968–1.031)	0.905	0.990 (0.959–1.022)	0.542
CI	0.730 (0.517–1.031)	0.074	0.771 (0.549–1.083)	0.134

LVEF: left ventricular ejection fraction; CI: cardiac index; HFrEF: heart failure with reduced ejection fraction; CV: cardiovascular; HF: heart failure.

^†^ Results are presented as risk ratio (95% confidence interval).

In the combined group of 65 HFmrEF and 73 HFpEF patients, neither LVEF nor CI was individually predictive of all-cause death or CV death. LVEF had no correlation with HF-readmission; however CI showed a significant association with HF-readmission. On adjusted analysis, CI proved to be an independent predictor of HF-readmission but not of all-cause or CV death, while LVEF had no independent predictive value for any endpoint (Table 5).

**Table 5:** Results of the adjusted and unadjusted multivariate Cox analysis in patients with HFmrEF and HFpEF.

	Unadjusted		Adjusted	
	Risk ratio^†^	*P—*value	Risk ratio^†^	*P—*value
All-cause death				
LVEF	1.010 (0.983–1.038)	0.470	1.010 (0.980–1.042)	0.514
CI	1.108 (0.819–1.501)	0.506	1.076 (0.806–1.437)	0.620
CV death				
LVEF	0.998 (0.957–1.040)	0.909	1.004 (0.960–1.049)	0.878
CI	1.011 (0.634–1.611)	0.964	1.021 (0.649–1.601)	0.930
HF-readmission				
LVEF	0.996 (0.966–1.027)	0.780	0.996 (0.962–1.031)	0.816
CI	0.653 (0.428–0.998)	0.049	0.634 (0.416–0.967)	0.034

LVEF: left ventricular ejection fraction; CI: cardiac index; HFmrEF: heart failure with mid-range ejection fraction; HFpEF: heart failure with preserved ejection fraction; CV: cardiovascular; HF: heart failure.

^†^ results are presented as risk ratio (95% confidence interval)

## Discussion

Our study fills a gap in information on echocardiographic measures of cardiac function. Left ventricular ejection fraction is used routinely as standard for the diagnosis and management of HF, however the moderate accuracy of the LVEF measurement raises concerns when using cut-off values as indication for potentially life-saving treatment.[12] Earlier recommendations for use of CI as an alternative measure were extrapolated from invasive examination, but studies confirming those results with echocardiographic measurement have been lacking.[10] The current analysis confirms that CI has predictive accuracy at least equal to LVEF in HF, thus CI may be a useful alternative to LVEF in cases in which the assessment of LVEF is challenging.

In contrast to earlier invasive studies of younger patients showing a strong association among LVEF, CI, and prognosis, our results show that both LVEF and CI measured by transthoracic echocardiography are poor predictors of all-cause death in an elderly HF cohort, which may be explained by a high proportion of deaths from causes other than HF in an aging population.[13, 14] Cardiac-index-based predictive models were found to be statistically more accurate than LVEF-based models, however the absolute difference was marginal.

The major limitation of this study was the timing of the echocardiography. A significant proportion of patients were examined early after admission for new onset HF, before initiation of therapy. As therapy may lead to an improvement in hemodynamic status and significant increase in both LVEF and CI, assessment of these parameters early in the disease course may have had limited value as predictors of later events.[15] Further prospective studies in a more homogenous clinical scenario are needed to overcome this limitation.

It is well known that approximately 30–40% of patients with guideline-based indication for cardiac resynchronization therapy do not experience improvement in left ventricular function or in clinical symptoms.[16, 17] Appropriate implantable cardioverter defibrillator (ICD) shocks are delivered in only a minority of ICD patients, thus most ICD implants do not result in clinical benefit.[18] Although the current study was not designed to draw assumptions of treatment indications, CI may be worth of consideration when predicting who will or will not respond to given therapies. Further research is needed to adequately assess the role of CI in the refinement of treatment indications in HFrEF.

The group of HF patients with an LVEF ≥40% has been the focus of research in recent decades. Results of all large mortality trials in this patient population have been neutral despite efforts to refine the diagnostic criteria for HF.[19] Our analysis suggests that CI may be a potential tool in the assessment of HFmrEF and HFpEF, and might be a useful selection criterion in future studies.

## Supporting information

S1 File Dataset. (XLSX)
